# Intravoxel Incoherent Motion Diffusion for Identification of Breast Malignant and Benign Tumors Using Chemometrics

**DOI:** 10.1155/2017/3845409

**Published:** 2017-05-29

**Authors:** Fengnong Chen, Pulan Chen, Hamed Hamid Muhammed, Juan Zhang

**Affiliations:** ^1^College of Life Information Science & Instrument Engineering, Hangzhou Dianzi University, Hangzhou 310018, China; ^2^School of Technology and Health, KTH Royal Institute of Technology, 14152 Stockholm, Sweden; ^3^Department of Economic Management, Cancer Affiliated Hospital of Xinjiang Medical University, No. 789 Suzhou East Road, Urumqi 830011, China; ^4^Zhejiang Cancer Hospital, Hangzhou 310022, China

## Abstract

The aim of the paper is to identify the breast malignant and benign lesions using the features of apparent diffusion coefficient (ADC), perfusion fraction *f*, pseudodiffusion coefficient *D*^⁎^, and true diffusion coefficient *D* from intravoxel incoherent motion (IVIM). There are 69 malignant cases (including 9 early malignant cases) and 35 benign breast cases who underwent diffusion-weighted MRI at 3.0 T with 8* b*-values (0~1000 s/mm^2^). ADC and IVIM parameters were determined in lesions. The early malignant cases are used as advanced malignant and benign tumors, respectively, so as to assess the effectiveness on the result. A predictive model was constructed using Support Vector Machine Binary Classification (SVMBC, also known Support Vector Machine Discriminant Analysis (SVMDA)) and Partial Least Squares Discriminant Analysis (PLSDA) and compared the difference between them both. The *D* value and ADC provide accurate identification of malignant lesions with *b* = 300, if early malignant tumor was considered as advanced malignant (cancer). The classification accuracy is 93.5% for cross-validation using SVMBC with ADC and tissue diffusivity only. The sensitivity and specificity are 100% and 87.0%, respectively, *r*^2^_cv_ = 0.8163, and root mean square error of cross-validation (RMSECV) is 0.043. ADC and IVIM provide quantitative measurement of tissue diffusivity for cellularity and are helpful with the method of SVMBC, getting comprehensive and complementary information for differentiation between benign and malignant breast lesions.

## 1. Introduction

Breast cancer is the most prevalent cancer among women worldwide. However, current imaging approaches (such as mammography) often do not provide enough information for proper lesion management, which sometimes results in unnecessary invasive treatments. Magnetic resonance imaging (MRI) and measurements of the apparent diffusion coefficient (ADC) have proven useful in the detection and characterization of cancer [1]. The ADC is sensitive to tissue cellularity and is usually lower in malignant tumors, in which water diffusion is more restricted because of the increased cell density and reduced extracellular space compared to the normal tissue. DW images may also reflect perfusion effects, as the microscopic blood flow in a randomly oriented capillary network creates a pseudodiffusion contribution to the DW signal.

DWI (diffusion-weighted imaging) is a functional magnetic resonance imaging (fMRI) of noninvasive examination; it can directly reflect the water molecule's Brownian motion in body's tissues. It can obtain physiological characteristics in body's tissues based on the quantitative analysis of water molecule's apparent diffusion coefficient (ADC). DWI has been widely used clinically, and it is through monoexponential model to calculate the ADC value, which contains two kinds of information of microcirculation perfusion and the water molecule diffusion of the organization. Therefore, ADC value of monoexponential model has been overestimated due to the microcirculation perfusion and it does not really reflect microstructure change of organization. In 1986, Le Bihan et al. [2] separated microcirculation perfusion and water molecule diffusion within the organization using biexponential model, to calculate separately perfusion fraction *f*, water molecule diffusivity (*D*, Slow ADC), pseudodiffusion (*D*^*∗*^, Fast ADC), and total apparent diffusion coefficient (ADC-total). In recent years, intravoxel incoherent motion (IVIM) has been widely used in a variety of well-vascularized tissues: they are head and neck [2–5], nose pharynx [6], lung [7, 8], liver [9, 10], kidney [11, 12], cervical [13], prostate [14], and the like. IVIMs are positively studying tumors in these aspects of blood perfusion, the identification between benign and malignant, scope of infringement, and curative effect evaluation.

The research of breast cancer has long been a contentious issue (*b* = 3~2500 s/mm^2^) over the number and selection of* b*-value as ADC rely highly on selection of* b*-value when scanning for IVIM. If* b*-value is between 0 and 200 s/mm^2^, IVIMs represent the information of microcirculation perfusion; the initial slope of perfusion fraction is counted by* b*-value, which between 0 and 100 s/mm^2^; with the increase of* b*-value, the ADC for sensitivity of perfusion is decrease; therefore,* b*-value selection should make little contribution to perfusion. Some researchers [15–20] believe that *D*^*∗*^ for the contribution to signal strength is very little when* b*-value is larger than 200 s/mm^2^;* D* represent pure diffusion, almost all of researchers get the same result that signal attenuation of malignant is more quick than benign and normal gland; and *D* and *f* play an important role in malignant and benign identification. Moreover, *D* is more sensitive than ADC (*b* = 0 and 1000 s/mm^2^), but *D*^*∗*^ make little sense to identification of malignant and benign tumor. When* b*-value is between 200 and 1000 s/mm^2^, IVIM represents diffusion information of water molecule; if* b*-value is larger than 1000 s/mm^2^, DKI (diffusion kurtosis imaging) reflects the non-Gauss diffusion movement of water molecule; for this reason, some researchers choose large-scale* b*-value model (*b* = 0~2500 s/mm^2^) and non-Gauss diffusion model. Iima et al. [15] and Suo et al. [17] use large-scale* b*-value model as well as* b*-value > 200 s/mm^2^ to identify malignant and benign tumor; for the former, the result shows that the ADC0 (apparent diffusion coefficient of diffusion kurtosis imaging) in malignant lesions was significantly lower than that in benign lesions and normal tissue, below tradition ADC value, too. ADC0 and *D* are significantly high to identify benign and malignant, which is similar to most of researchers with biexponential model of IVIM; besides, another non-Gauss parameter of diffusion kurtosis model, mean kurtosis, is added to identification. For the latter, the result shows that the parameter of IVIM relied on different mathematical computing according to the comparison of 3 different* b*-values. Therefore, in order to evaluate the effect with different* b*-value, in this study, we choose 3* b*-values (150, 200, and 300) to test.

Along with* b*-value increase, the diffusion time of water molecule is extended gradually; at the same time, the clinic examination time will extended. At present, the lack of standard and optimization in* b*-value selection gives rise to several problems; little is known about clinical significance of different* b*-value parameter between 200 and 1000 s/mm^2^. Also, the reliability of the IVIM measurements achievable in clinical practice and their usefulness in cancer diagnosis need to be further evaluated. The purpose of this study was to use DW MRI at 3.0 T and

1. to extract parameters corresponding to different* b*-value in biexponential model;
2. to find out the clinical significance of benign and malignant tumor identification based on big* b*-value of biexponential model in IVIM;
3. to assess the ability of the IVIM parameters and ADC to differentiate malignant lesions from benign lesions and, furthermore, to compare the difference of identification between two conditions, which are whether the early malignant is regarded as cancer or not.

## 2. Materials and Methods

### 2.1. Patient Selection

This is a retrospective study; therefore, Ethics Committee agreed to give informed consent. Based on our selection criteria,* 78 patients* were identified and their MRI studies were reviewed by an experienced radiologist and pathologist who had access to all patient information and analyzed the biopsy specimens and identified the tumor histological type as well as the tumor histological grade and nuclear grade. Between March and November in 2015, a total of 78 patients (mean age: 48.9 years; range: 15–70 years) with MRI (including multi-*b*-value DWI) and dynamic contrast-enhanced (DCE) were collected in this study; all patients were first to see doctor and no treatment is performed. In every patient, a single largest lesion in each breast was selected; examination revealed 72 positive patients and 6 cases with normal glands, of which 98 lesions were found, including 60 advanced invasive ductal carcinomas (IDC), 9 ductal carcinomas in situ (DCIS), and 29 benign lesions (including 7 cysts, 6 fibroadenomas, 1 hamartoma, 1 intraductal papilloma, 5 adenoses of breast, and 9 apocrine metaplasia cases). Lesions were excluded if their in-plane dimensions were smaller than 8 mm or if their diffusion-weighted MR images contained artifacts, such as poor fat suppression or susceptibility artifacts from biopsy and surgical clips. The final diagnoses are as follows: all malignant tumors were confirmed on the basis of histopathology and immunohistochemistry. The 6 normal glands were confirmed based on magnetic resonance imaging and 7 cysts were confirmed based on ultrasonic, mammography according to BI-RADS (breast imaging reporting and data system) of assessing mode. The left benign tumors were confirmed by surgery and pathology.

### 2.2. MR Image Acquisitions

MR imaging was performed by a 3.0-T MR imager (Siemens 3.0 T, Siemens Verio 3.0, Germany) equipped with a 16-channel SENSE breast coil in prone position. All patients are fasted 6 hours before examining, and we checked both breasts at the same time with prone position of head first. MRI routine scan parameter was as folllows: (1) T2-TSE-TRA-FS: TR 5800 ms, TE 84 ms, flip angle 150.0, slice thickness 5.0 mm, slice gap 6.0 mm, FOV 320 mm *∗* 320 mm, reconstruction matrix 320 mm *∗* 224 mm, and scan time 1 min 33 seconds; (2) T1-TSE-TRA: TR 704 ms, TE 10.0 ms, flip angle 150.0, slice thickness 5.0 mm, slice gap 6.0 mm, FOV 320 mm *∗* 320 mm, reconstruction matrix 320 mm *∗* 224 mm, and scan time 39 seconds; (3) 2d EPI-diff-IVIM: TR6600 ms, TE 67.0 ms, flip angle 90, slice thickness 5.0 mm, slice gap 6.5 mm, FOV 350 mm *∗* 188 mm, reconstruction matrix 112 mm *∗* 60 mm, 8* b*-values: 0, 50, 100, 150, 200, 300, 400, 800, and 1000 s/mm^2^,* b*-values being carried out in the diffusion gradient direction of *X*, *Y*, *Z* with 3 times of excitation, and scan time 5 min and 50 seconds; (4) DEC: T1-fl3d-TRA-FS: TR4.5 ms, TE1.6 ms, flip angle 10, slice thickness 1.2 mm, slice gap 1.2 mm, FOV340 mm *∗* 340 mm, and reconstruction matrix 448 mm *∗* 300 mm.

Contrast material is required after precontrast (about 20 seconds delay) and 5 consecutive time points after administration of gadolinium (Gd-DTPA, 25 mL) by high pressure injector. After that, 25 mL normal saline was injected as well with the injection speed of 2.0 mL/s; the duration lasted for 4 min 57 seconds.

### 2.3. MRI Analysis

**Figure 1:**
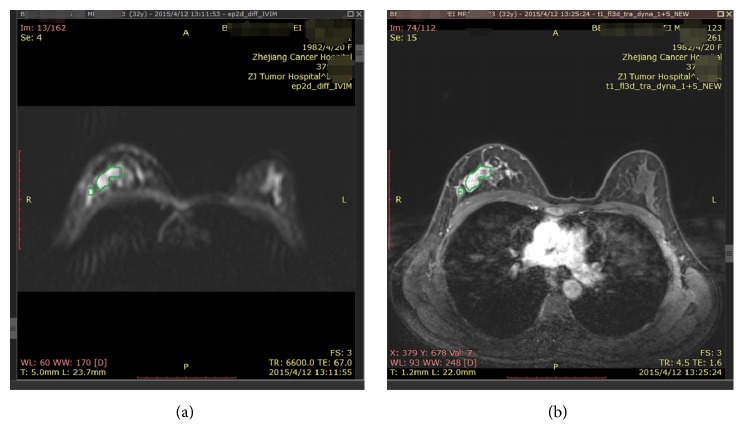
IVIM (a) and DCE-T3 (b) images of patient case.

The accuracy of ADC is closed related to experience of observer other than the region of ROI [21]; the IVIM image is not clearly compared to DCE (Figure 1), so to get reliable results, in this study, 25-year and 5-year experienced radiologists read the MRI database, to determine the largest slices and the largest substantial tumor in MRI according to T1W and T2W at the exclusion of bleed, necrosis and cystic lesion, and edema region at first; then to get the region and scale of ROI, every case is determined by 3 ROIs; if the diameter of focus is less than 1.5 cm, only one ROI is used.

The IVIM features are got by an open-source software of MITK (German cancer research center, MITK diffusion 2014.10.02). IVIM analysis: the biexponential model from an IVIM sequence was expressed by the following equation, as described by Le Bihan et al. [22]:

(1) $$\begin{array}{c} \frac{Sb}{S0}=\left(\;1-f\;\right)\exp\left(\;-b\times D\;\right)+f\exp\left[\;-b\times \left(\;D+D^{*}\;\right)\;\right], \end{array}$$

where *Sb* is the signal intensity in the pixel with diffusion gradient* b*, *S*0 is the signal intensity in the pixel without diffusion gradient, *D* is the true diffusion as reflected by pure molecular diffusion, *f* is the fractional perfusion related to microcirculation, and *D*^*∗*^ is the pseudodiffusion coefficient representing perfusion-related diffusion or incoherent microcirculation. To get consecutive IVIM parameter, different* b*-values are chosen, which are 150, 200, and 300 s/mm^2^; the three parameters were calculated consecutively in which *D* was obtained by a simplified linear fit equation (*Sb* = *S*0 × exp⁡(−*bD*)) when* b*-values are larger than 200 s/mm^2^. This was based on the assumption that *D*^*∗*^ is significantly greater than *D* such that its influence on signal decay can be neglected for* b*-values > 200 s/mm^2^. *f* and *D*^*∗*^ were calculated by using a nonlinear regression algorithm for all* b*-values.

Parameters mapping is got by loading IVIM into MITK. The choice of ROI is controversial; some researchers chose ROI according to the level of maximum transverse diameter of lesions [18]. But small ROIs show less overlap in ADC values and higher ADC reproducibility, suggesting that this method may improve lesion discrimination. Interobserver variability was low for both methods [20]. Therefore, in this study, ROI was manually placed on each lesion using small ROIs, consistent with minimal contaminations from surrounding unintended tissues. The value of ROI is an average from 3 ROIs so as to get more reliable value. ADC values were measured on ADC maps produced by equation (ADC = In⁡(*S*1/*S*2)/(*b*2 − *b*1)) from* b* = 0 and 1000 s/mm^2^ using client software of Siemens and the ROIs were kept as close as possible to those on IVIM parametric maps. For the contralateral healthy breast tissues, the sizes of ROIs were in a range of 10~25 pixel points and excluded large vessels and ducts. *D*, *f*, *D*^*∗*^, and ADC values were measured by two independent observers with experiences of 5 and 25 years (the author of this manuscript: Juan Zhang) of breast MRI diagnosis.

The features detailed description of IVIM are as follows: *D* (the true diffusion as reflected by pure molecular diffusion), *f* (the fractional perfusion related to microcirculation), and *D*^*∗*^ (the pseudodiffusion coefficient representing perfusion-related diffusion or incoherent microcirculation). Those IVIM features can be obtained from the software of MITK. Also, the lists of features and the explanation were given in Table 1. The image and data analyses package were developed by MATLAB (Version 7.9, The Mathworks Inc., Natick, MA).

### 2.4. Dataset Construction and Preprocessing

There are several methods that have been developed for predicting and identifying, such as Fang et al.'s study [23], which used feature selection algorithms to identify 16 features, out of a total of 560 physicochemical properties, presumably important to protein aggregation. Two predictors (ProA-SVM and ProA-RF) using selected features are built for predicting peptide aggregation propensity and identifying aggregation prone regions in proteins. Both methods are compared favorably to other state-of-the-art algorithms in cross-validation. We can gain a great deal of enlightenment from the article.

In this paper, several steps are carried out for analysis, which consist of normalization, ROC analysis, identification method description, and the result of identification. All statistical tests were conducted at the two-sided 5% significance level using MATLAB 2014 and SPSS 19.

#### 2.4.1. Normalization

At first, it was necessary to scale the dataset. The main advantage of scaling the dataset was to avoid attributes in greater numeric ranges dominating those in smaller numeric ranges. Numerically, a variation in ADC between 300 and 500 is much greater than a variation in *d*^*∗*^ between 0.01 and 0.1. However, the effect of each of these variables on the system of interest may be very similar. For that reason, it may be advisable to scale the data.

Another advantage was to avoid numerical difficulties during the calculation. Also, our experiments have shown that feature value scaling could increase the accuracy. Generally, each feature can be linearly scaled to the range [−1, +1] or [0, 1]. In this work, we chose the range [0, 1] by the following formula:

(2) X−=1n∑i=1nxiS=1n−1 ∑i=1nxi−X−2Fx=xi−X−S,

where *x* is the original feature, *S* is the standard deviation, and *F* is the final value of normalization.

#### 2.4.2. ROC Analysis

Region of concern (ROC) analyses was used to assess the diagnostic utility, for the detection of malignant lesions or lesions characterized as positive for a given marker. The area under the ROC curve (AUC) was used to assess the diagnostic utility for the detection of lesions characterized as positive as well. Sensitivity, specificity, and overall accuracy were computed at the threshold value of each measure that maximized the Youden index in an ROC analysis.

Here, sensitivity (also called the true positive rate, TPR) and specificity (also called the true negative rate, TNR) are statistical measures of the performance of a binary classification test, also known in statistics as classification function; the Youden index (sensitivity + specificity − 1) is a frequently used summary measure of the ROC curve. It, both, measures the effectiveness of a diagnostic marker and enables the selection of an optimal threshold value (cutoff point) for the marker; Matthews correlation coefficient (MCC) is also an important index, expressed by (3). It takes into account true and false positives and negatives and is generally regarded as a balanced measure which can be used even if the classes are of very different sizes. The MCC is in essence a correlation coefficient between the observed and predicted binary classifications; it returns a value between −1 and +1. A coefficient of +1 represents a perfect prediction, 0 is no better than random prediction, and −1 indicates total disagreement between prediction and observation:

(3) MCC=TP×TN−FP×FNTP+FPTP+FNTN+FPTN+FN,

where TP is the number of true positive samples; TN is true negative; FN is the number of false negative samples; FP is the number of false positive samples; and TP is the number of true positive samples.

#### 2.4.3. Identification Methods

In order to get more accuracy of benign and malignant identification, several methods are used to try to get the best method. This study makes an attempt on combining chemometrics and cancer identification.

Chemometrics methods can highlight the chemical differences between samples and reduce variation due to physical effects. The combination of cancer features and chemometrics methods was investigated for qualitative analysis. Multivariate analyses including PLSDA and SVMBC proven to be effective in many applications [24] were used in the present study to classify benign and malignant tumor with different features. The success of these methods depends on the choice of proper case and the number of variables employed in the calibration model.

*PLSDA*. In this study, ADC and IVIM features were used to establish models in PLSDA for the discriminant analysis of benign and malignant tumors. Each case was assigned a dummy variable 1 or 2 as a reference value for the class labels; the prediction result will indicate whether the sample belongs to a particular group or not [25]. Here, malignant samples were assigned a numeric value of 2, and those of benign assigned 1. After assigning the reference value for each case, the PLSDA model was then developed. If the predicted values lay on the same side of the threshold (mid value between two labels normally) of the assigned values, the case was considered to be correctly categorized [26]. If the predicted value was between 0.5 and 1.5, the benign tumor sample was classified correctly; else the sample was classified as wrong. Similarly, if the predicted value was between 1.5 and 2.5, it was malignant tumor sample [25]. It is expected to have ideal models with the lower root mean square error of cross-validation (RMSECV), and the higher correlation coefficient of calibration and cross-validation, *R*_*c*_ and *R*_cv_, respectively [27]. 

*SVMBC*. SVM is a supervised learning model with associated learning algorithms that analyze data used for classification and regression analysis, was introduced by Cortes and Vapnik in the late 1960s on the foundation of statistical learning theory [28]. It is a way to create nonlinear classifiers by applying the kernel trick to maximum-margin hyperplanes. The optimal separating hyperplane is determined by giving the largest margin of separation between different classes. For the two-class (binary classification, just for malignant and benign discrimination) case in SVM model, this optimal hyperplane bisects the shortest line between the convex hulls of the two classes.

*Cross-Validation*. The last step for estimating the prediction error is cross-validation, which is a model validation technique for assessing how the results of a statistical analysis will generalize to an independent dataset, and one wants to estimate how accurately a predictive model will perform in practice. Leave-one-out cross-validation (LOOCV) is a common method to do so. In this validation, all cases except one are used to construct a model; the remained cases are used to predict. This is repeated on all ways to cut the original case on a validation set. The advantages of cross-validation are that all of the test cases were independent and the reliability of the results could be improved. The dataset is divided into two subsets for cross-validation.

## 3. Result and Discussion

Previous studies had demonstrated that ADC and *D* value are very useful in the differential diagnosis of breast lesions. In this study, Receiver Operating Characteristic curves, with statistics were calculated for ADC and IVIM features under the condition of 3 *b*-value.

The ROC analyses to assess diagnostic utility for the detection of malignant lesions reveals that the average ADC and *D* values had higher AUC values (0.942 and 0.921, resp.), Youden index (0.7839 and 0.7834, resp.), and Matthews correlation coefficient (0.7579 and 0.7493, resp.) when *b*-value = 300. It is obvious that ADC and *D* on *b* = 300 contribute to the identification of malignant and benign tumor (Table 2).

From Table 2, we obtained similar results to the other researchers. While the AUC values for *D* and ADC were not significantly different, *f* and *D*^*∗*^ values showed a lower AUC than those of ADC and *D* value.

However, the difference is not very obvious among 3* b*-values based on ADC and *D*, in order to get more detailed results; in the next step, we try to analyze early malignant tumor and cancer separately using chemometrics, which is applied to solve both descriptive and predictive problems in experimental natural sciences.

### 3.1. Chemometrics Analysis

In many cases, it is very necessary to find early malignant tumor. The sooner the cancer is diagnosed and treated, the better the person's chance is for a full recovery. In its early stages, soft tissue malignant tumors rarely cause any symptoms. Because soft tissue is very elastic, the tumors can grow quite large before they are felt. The first symptom is usually a painless lump. As the tumor grows and begins to press against nearby nerves and muscles, pain or soreness can occur.

As we know, early malignant tumor is difficult to recognize and the treatment has been highly effective the general prognosis. So, in this study, early malignant tumors are used as different group to analyze; firstly, early malignant cases are as malignant tumor together with advanced malignant tumor; secondly, early malignant cases are analyzed as benign tumor cases. There are 60 malignant tumor cases, 9 early malignant tumor cases, and 35 benign tumor cases. That means, at first, the number of malignant cases is 69 and benign is 35; secondly, the number of malignant cases is 60 and benign is 44.

Besides early malignant cases, IVIM features also are disputed. Cho et al. [29] conclude that the average values of the ADC and IVIM biomarkers, tissue diffusivity, and perfusion fraction showed significant differences between benign and malignant lesions. Liu et al. [19] believe that tissue diffusivity and ADC values demonstrated higher sensitivity and specificity in differentiating benign lesions and malignant tumors. So, we try to use different features combination to get the best result. Firstly, ADC and all IVIM features (including *f*, *D*, *D*^*∗*^) are taken as input to build model and predict, and, then, the input features are replaced with ADC and tissue diffusivity to deal with them again.

Another question is* b-*value; as opinions vary, no unanimous conclusion can be drawn. Here, we try to use 3* b*-values (*b* = 50,200,300 s/mm) to assess the effectiveness of benign and malignant identification.

### 3.2. Identification

#### 3.2.1. Early Malignant Analysis as Advanced Malignant

In this section, there are two methods to identify those cases with different* b*-value, which are PLSDA and SVMBC. Each method processes data with two different input features, one is ADC and 3 IVIM features, and another is ADC and tissue diffusivity. 

*PLSDA*.  Table 3 is the result of tumor analysis using PLSDA models on the ADC and IVIM features with different *b*-values (150, 200, and 300) under the condition that early malignant cases are considered as advanced malignant. The results consist of several parts, the analysis steps include calibration, cross-validation, and prediction, and the evaluation items comprise sensitivity, class error, RMSE, and correlation. Among other things, the sensitivity of benign and malignant is a pair of relative quantity, if the index of benign is sensitivity, which is also the specificity of malignant. Likewise, the sensitivity of malignant is the specificity of benign. The results indicate that no matter which data treatment it is, the results are the best when* b*-value is 300 for sensitivity, specificity, and accuracy at the stage of calibration and cross-validation with 0.870, 0.978, and 0.0761. Besides, the correlation RMSEC and RMSECV are also good in performance with high correlation coefficient and low root mean square error. But for prediction, the result is the best when the select* b*-value is 200 for sensitivity, specificity, class error, and RMSEP. However, the correlation coefficient is low. Therefore, it is necessary to balance calibration, cross-validation, and prediction; otherwise, it is difficult to find a best method. 

*SVMBC*.  Table 4 shows that the best result is unclear. Although the sensitivity of malignant, class error, RMSECV, and correlation are the best among three* b*-values, the sensitivity of benign is the lowest, only 0.565. 

*Using ADC and Tissue Diffusivity Only*. Because some researchers believe that just ADC and tissue diffusivity are the most useful features, so, here we try to use only 2 features to analysis those data.  Table 5 shows the best results of sensitivity and specificity are 0.87 and 0.978, respectively; the result is the same as before that the input features are ADC and 3 IVIM features. 

*SVMBC*.  Table 6 shows that when* b*-value is 300, no matter which index, it can get the best result; the sensitivity of benign and malignant cases is 0.87 and 1, the accuracy is 93.48%, the RMSECV is 0.0435, the correlation is 0.8163, so, and the result is ideal.

#### 3.2.2. Early Malignant Analysis as Benign Malignant

In order to prove the influence of early tumor on the result, early tumor is regrouped as benign tumor and reanalyzed once using the same methods above. Tables 7–10 show the result of PLSDA, which is similar to that of Section 3.2.1. There is no obvious difference between them. For SVMBC, see Tables 8 and 10; for using ADC and tissue diffusivity only, see Table 9.

In conclusion, the difference between advanced malignant and benign cases with early tumor is subtle. Table 1 is the statistics for benign and malignant cases using PLSDA models on the IVIM and ADC features with different treatments. The results indicate that SVMBC can improve accuracy when classifying early malignant tumor as advanced malignant, compared with another* b*-value; the ADC and tissue diffusivity with* b* = 300 had the best results, where *r*_*c*_ = 0.8163, the sensitivity is 1 (I think it is happened to get), specificity is 0.870, RMSECV is 0.0435, and accuracy is 93.5%.

In this study, there are several limitations; the first is the biased patient cohort with a small range of diseases types, which may obscure the identification of benign and malignant cases. Then, the number of* b*-values' selections for IVIM is still unknown; how many *b* values and which one or ones are suitable? Thirdly, there are just 4 features (3 IVIMs and ADC); it is difficult to extract useful features to identify among them.

## 4. Conclusion

This study shows that differences between benign and malignant tumor do exist and groups are apparent. ADC and IVIM combined with multivariate analysis have been proved to be a very powerful tool for judgment of the relative pattern of the objects that have very similar properties. Like ADC value, *D* also can be used to differentiate benign and malignant lesions and had the highest specificity. Combining with *f* or *D*^*∗*^ value, *D* value can increase diagnostic sensitivity and may have a vital role in screening breast MRI in high-risk women.

The results of this study show that an excellent classification can be obtained by SVMBC, with accuracy about 100% (the accuracy is very high in this method as the reason of sample data selection; I think, in order to get more reliable result, it should collect much more cases.). The methods got comprehensive and complementary information to distinguish benign and malignant tumors. Further studies are needed to use more parameters expressing features of tumor and more discriminant analysis methods to develop valuable and robust models to discriminate other tumors.

## Figures and Tables

**Table 1:** Extracted features of individual tumor region.

Number	Code	Feature explanation	Formula or description
1	*D*	Tissue diffusivity	IVIM features
2	*f*	Perfusion fraction	
3	*D* ^*∗*^	Pseudodiffusion coefficient	
4	ADC	Apparent diffusion coefficient	Measure of the magnitude of diffusion

**Table 2:** The ROC results of IVIM and ADC.

	*b*-value	AUC	Std. error	Asymptotic Sig.	Youden index	MCC
	*ADC*	*0.942*	*0.029*	*0.000*	*0.7839*	*0.7579*
*B* = 150	*f*	0.291	0.055	0.001	0	0
	*D*	0.918	0.033	0.000	0.7545	0.7178
	*D* ^*∗*^	0.415	0.064	0.157	0.0559	−0.1562
*B* = 200	*f*	0.308	0.057	0.001	0.0137	−0.33
	*D*	0.918	0.033	0.000	0.7545	0.7178
	*D* ^*∗*^	0.414	0.064	0.154	0.0828	−0.21
*B* = 300	*f*	0.297	0.055	0.001	0.0282	−0.1383
	*D*	*0.921*	*0.033*	*0.000*	*0.7834*	*0.7493*
	*D* ^*∗*^	0.337	0.061	0.007	0	0

**Table 3:** Results of *PLSDA* for tumor analysis with *ADC  and  IVIM  features  *(early malignant as advanced malignant).

Data treatment	*b*-value	Sensitivity		Class. Err	RMSE	*R* ^2^
		Benign	Malignant			
Calibration	150	0.783	0.935	0.1413	0.3263	0.5207
	200	0.783	0.978	0.1196	0.3266	0.520
	*300*	*0.870*	*0.978*	*0.0761*	*0.2809*	*0.6450*
Cross-validation	150	0.739	0.935	0.1630	0.3536	0.4451
	200	0.696	0.978	0.1630	0.3612	0.4285
	*300*	*0.870*	*0.978*	*0.0761*	*0.3100*	*0.5712*
Prediction	150	0.917	0.870	0.1069	0.3532	0.5676
	200	0.917	0.870	0.1069	0.276	0.01565
	300	0.583	0.826	0.29529	0.3989	0.3024

**Table 4:** Results of *SVMBC* for tumor analysis with *ADC and IVIM features* (early malignant as advanced malignant).

Data treatment	*b*-value	Sensitivity		Class. Err	RMSE	*R* ^2^
		Benign	Malignant			
Cross-validation	150	0.826	0.978	0.0978	0.05797	0.69
	200	0.565	1	0.2174	0.08696	0.4642
	300	0.783	1	0.01087	0.05797	0.7058

*Note*. Here, some results are 100% in this model; (the same as in Tables 5–10).

**Table 5:** Results of PLSDA for tumor analysis with *ADC and tissue diffusivity* (early malignant as advanced malignant).

Data treatment	*b*-value	Sensitivity		Class. Err	RMSE	*R* ^2^
		Benign	Malignant			
Calibration	150	0.739	0.935	0.1630	0.3279	0.5160
	200	0.783	0.978	0.1196	0.3337	0.4987
	*300*	*0.87*	*0.978*	0.076	0.2809	0.6449
Cross-validation	150	0.739	0.935	0.1630	0.3494	0.4574
	200	0.739	0.957	0.1522	0.3602	0.4258
	300	*0.87*	*0.978*	*0.076*	0.2989	0.59972
Prediction	150	0.917	0.652	0.2156	0.3556	0.5606
	200	*0.917*	*0.783*	0.1504	0.3313	0.6095
	300	0.583	0.826	0.2952	0.3996	0.2999

**Table 6:** ADC and tissue diffusivity.

Data treatment	*b*-value	Sensitivity		Class. Err	RMSE	*R* ^2^
		Benign	Malignant			
Cross-validation	150	0.652	0.913	0.2174	0.075	0.3558
	200	0.870	0.891	0.1196	0.0725	0.5568
	*300*	*0.870*	*1*	*0.0652*	*0.043478*	*0.8163*

**Table 7:** Results of PLSDA for tumor analysis with *IVIM* (early malignant as benign tumor).

Data treatment	*b*-value	Sensitivity		Class. Err	RMSE	*R* ^2^
		Benign	Malignant			
Calibration	150	0.739	0.950	0.15544	0.33135	0.5264
	200	0.783	0.900	0.1587	0.3417	0.4963
	*300*	*0.870*	*0.925*	0.1027	0.3212	0.5549
Cross-validation	150	0.739	0.900	0.18044	0.377528	0.4088
	200	0.696	0.900	0.2022	0.4063	0.3421
	300	*0.783*	*0.900*	*0.2655*	0.3564	0.4613
Prediction	150	0.714	0.900	0.1929	0.3800	0.4616
	200	*0.714*	*0.900*	0.1929	83.6087	0.0274
	300	0.619	0.850	0.2655	0.4154	0.3915

**Table 8:** ADC and IVIM features.

Data treatment	*b*-value	Sensitivity		Class. Err	RMSE	*R* ^2^
		Benign	Malignant			
Cross-validation	150	0.739	0.900	0.1804	0.0952	0.4261
	200	0.565	0.900	0.2674	0.1111	0.2546
	*300*	*0.826*	*0.925*	*0.1245*	*0.1270*	*0.5754*

**Table 9:** Results of PLSDA for tumor analysis with tissue diffusivity (early malignant as benign tumor).

Data treatment	*b*-value	Sensitivity		Class. Err	RMSE	*R* ^2^
		Benign	Malignant			
Calibration	150	0.783	0.925	0.1462	0.3338	0.5193
	200	0.826	0.950	0.1120	0.3538	0.4600
	*300*	*0.696*	*0.900*	0.2022	0.3373	0.5091
Cross-validation	150	0.783	0.925	0.1462	0.3659	0.4379
	200	0.783	0.900	0.1587	0.3926	0.3605
	300	*0.696*	*0.900*	*0.2022*	0.3540	0.4615
Prediction	150	0.762	0.900	0.1690	0.375	0.4730
	200	*0.762*	*0.950*	0.1440	0.3666	0.4977
	300	0.667	0.900	0.2167	0.4035	0.4672

**Table 10:** ADC and tissue diffusivity features.

Data treatment	*b*-value	Sensitivity		Class. Err	RMSE	*R* ^2^
		Benign	Malignant			
Cross-validation	150	0.913	0.825	0.1310	0.0794	0.5114
	200	0.870	0.875	0.1277	0.0794	0.5369
	*300*	*0.696*	*1*	*0.1522*	*0.09524*	*0.5920*
